# Absolute Cerebral Blood Flow Infarction Threshold for 3-Hour Ischemia Time Determined with CT Perfusion and ^18^F-FFMZ-PET Imaging in a Porcine Model of Cerebral Ischemia

**DOI:** 10.1371/journal.pone.0158157

**Published:** 2016-06-27

**Authors:** Eric A. Wright, Christopher D. d’Esterre, Laura B. Morrison, Neil Cockburn, Michael Kovacs, Ting-Yim Lee

**Affiliations:** 1 Department of Medical Biophysics, Western University, London, Ontario, Canada; 2 Robarts Research Institute, Western University, London, Ontario, Canada; 3 Lawson Imaging, Lawson Health Research Institute, London, Ontario, Canada; 4 Department of Radiology, Foothills Medical Center, University of Calgary, Calgary, Alberta, Canada; 5 Department of Medical Imaging, Western University, London, Ontario, Canada; 6 Department of Oncology, Western University, London, Ontario, Canada; Henry Ford Health System, UNITED STATES

## Abstract

CT Perfusion (CTP) derived cerebral blood flow (CBF) thresholds have been proposed as the optimal parameter for distinguishing the infarct core prior to reperfusion. Previous threshold-derivation studies have been limited by uncertainties introduced by infarct expansion between the acute phase of stroke and follow-up imaging, or DWI lesion reversibility. In this study a model is proposed for determining infarction CBF thresholds at 3hr ischemia time by comparing contemporaneously acquired CTP derived CBF maps to ^18^F-FFMZ-PET imaging, with the objective of deriving a CBF threshold for infarction after 3 hours of ischemia. Endothelin-1 (ET-1) was injected into the brain of Duroc-Cross pigs (n = 11) through a burr hole in the skull. CTP images were acquired 10 and 30 minutes post ET-1 injection and then every 30 minutes for 150 minutes. 370 MBq of ^18^F-FFMZ was injected ~120 minutes post ET-1 injection and PET images were acquired for 25 minutes starting ~155–180 minutes post ET-1 injection. CBF maps from each CTP acquisition were co-registered and converted into a median CBF map. The median CBF map was co-registered to blood volume maps for vessel exclusion, an average CT image for grey/white matter segmentation, and ^18^F-FFMZ-PET images for infarct delineation. Logistic regression and ROC analysis were performed on infarcted and non-infarcted pixel CBF values for each animal that developed infarct. Six of the eleven animals developed infarction. The mean CBF value corresponding to the optimal operating point of the ROC curves for the 6 animals was 12.6 ± 2.8 mL·min^-1^·100g^-1^ for infarction after 3 hours of ischemia. The porcine ET-1 model of cerebral ischemia is easier to implement then other large animal models of stroke, and performs similarly as long as CBF is monitored using CTP to prevent reperfusion.

## Introduction

Both MRI and CT are highly sensitive for infarct core. Generally, CT is used preferentially for stroke diagnosis/prognosis because of availability, cost and speed. Along with non-contrast CT and CT Angiography, CT Perfusion (CTP) is now consistently acquired at many institutes. CTP based time-dependent thresholds for infarct core have been recently derived using data from ischemic stroke patients [1]. These thresholds will have important implications for patient triaging, and will be useful in wake-up and late presenting strokes. Predicting the infarct core evolution could help identify patients who will benefit most from transfer to tertiary centers capable of intra-arterial therapy (IAT), the new standard of care.

However, many threshold derivation studies used follow-up imaging performed 1–7 days after symptom onset to define the infarct core [2–8], introducing uncertainty caused by infarct expansion in the time between admission and follow-up imaging. Furthermore, some of these studies used diffusion weighted imaging (DWI) to define the infarct core [5,6,8]. DWI lesion reversal has been observed in both human and animal ischemic stroke [9,10], though it should be noted that clinical instances of DWI lesion reversal are rare [11], and should not deter anyone from using MRI if it is logistically feasible to acquire in the acute setting.

One alternative, which may circumvent uncertainties caused by infarct expansion and DWI, is to use large animal stroke models to derive time-dependent thresholds for infarction. The logistical complexity of producing and using radiotracers in the clinical acute stroke setting are not a factor, and radiation dose is less of a concern in animal models, so the infarct can be defined using PET imaging with radiolabeled flumazenil (FMZ), or its fluorinated analog, fluoroethylflumazenil (FFMZ). This gold standard method reliably predicts the final infarct and is less prone to false positives then DWI [12]. Furthermore, animal models provide greater control over the time interval between symptom onset and tissue status determination, allowing infarction thresholds to be determined for many different ischemia durations.

Porcine models are more useful then small animal models since the gyrencephalic brain is more similar to a human brain in terms of grey/white matter composition and size [13]. However, the rete mirabelle makes it impossible to use intra-arterial catheter based methods commonly used to initiate cerebral ischemia in small animal models [14]. As a result, most porcine models of stroke rely on complex and invasive surgical procedures to access the middle cerebral artery so a clip or ligature can be applied [15,16]. Using endothelin-1 (ET-1) to cause transient cerebral ischemia does not require complicated surgical procedures and has been well established in rodents and lower primates [17,18]. Application of ET-1 induces changes in CBF that are severe enough to induce infarction, with minimal tissue edema. Recently, the ET-1 method was used in a porcine model of cerebral ischemia [19].

In this study, we presented an ET-1 based porcine model of cerebral ischemia for determining time dependent CBF thresholds for infarction using CTP and ^18^F-FFMZ-PET imaging, and we determined a CBF threshold for infarction after 3hrs of ischemia.

## Methods

### Acute Cerebral Ischemia Model

All animal experiments were conducted following the guidelines of the Canadian Council on Animal Care and approved by the Animal Use Subcommittee at the University of Western Ontario (Protocol #2007–050). Duroc Cross Pigs were picked up from a nearby farm on the day of the experiment and were not housed at the laboratory prior to experiments. Out of the 11 animals (average weight 26 ± 5 kg) used in this study, 7 were female and 4 were male. Anesthesia was induced in the animals using 4–5% isoflurane. Anesthesia was maintained by mask with 3–4% isoflurane until intubation. Propofol (16–22 mg/kg) was given IV for intubation. The pig was ventilated (10–15 cc/stroke volume, 20–30 breath per minute) with 2.5–3.5% isoflurane with oxygen and medical air (2:1 medical air to oxygen ratio) for the duration of the experiment. A 22G cephalic vein catheter was placed for injection of CT contrast (Isovue 370) and ^18^F-FFMZ. One femoral artery was cannulated with a catheter for measuring blood pressure, blood gases (pO_2_ and pCO_2_), glucose and pH throughout the experiment. In addition heart rate (HR), arterial oxygen saturation (S_a_O_2_), end-tidal carbon dioxide tension (CO_2_), respiration rate (RR) and blood pressure (BP) were continuously monitored (Surgivet). The animal was wrapped in a circulating hot water blanket and rectal temp were monitored continuously throughout the experiment.

A CT scan was done to identify sixteen contiguous 2.5mm thick slices which included the largest coronal sections of the brain, then a baseline CTP study was performed using the procedures outlined in the next section. A target slice location showing the maximal extent of the middle cerebral artery (MCA) territory was selected following the baseline CTP scan. This target slice was marked on the pig’s head using the CT scanner laser positioning light, the scalp was incised (silver nitrate sticks used to control bleeding) and a 1-2mm diameter burr hole was made with a Dremel hand tool through the skull. A 27G 1 ¼” long needle attached to a 1mL saline syringe with PE 40 tubing was preloaded with ET-1 and inserted through the burr hole into the brain. An axial CT scan was then acquired to verify that the needle tip is within the cerebral cortex in the target slice. 33μg of ET-1 in 150μL of sterile water was injected at 50 μL/min using an infusion pump. CT Perfusion studies were performed 10 and 30min after the ET-1 injection, and then every 30min for the remainder of the 3hr monitoring period.

Animals were under anesthetic for the entire study to reduce unnecessary animal suffering. If anything seriously detrimental had happened during surgery or scanning, the animal would have been euthanized immediately by intravenous potassium chloride overdose under full deep anesthetic, however there were no serious incidents so early termination of experiments was not necessary. At the conclusion of the experiment animals were euthanized by intravenous potassium chloride overdose under full deep isoflurane anesthetic.

### On-line CBF Monitoring with CT Perfusion

CTP studies were acquired on the GE Healthcare Discovery VCT PET/CT scanner using the following protocol: 80kV, 200mA, 16 slices of 2.5mm thickness, 1 scan per second for 60s with a 5s delay from the start of contrast injection (370mg Iodine/mL) at a dosage of 1mL/kg body weight and at an injection rate of 3mL/s. CTP studies were completed at baseline, 10 and 30min post ET-1 injection and then every 30min until 3hr post ischemia. Quantitative CBF maps from each acquired CTP study, calculated within 5min of acquisition, were used to evaluate the perfusion in the ET-1 injection territory. If reperfusion caused CBF to rise above the target range of ~20 mL·min^-1^·100g^-1^ (infarction threshold with permanent occlusion [20]), a second dose of ET-1 was injected and perfusion was checked 10min after, before perfusion monitoring went back to half-hourly intervals. Physiological parameters that have an impact on CBF were monitored throughout the experiment.

### ^18^F-FFMZ-PET Imaging for Detecting Cerebral Infarction

FMZ is a selective, high-affinity ligand for the central benzodiazepine receptor of the GABA-A receptor complex [21]. Since cortical neurons have a high concentration of GABA-A receptors, and they are sensitive to early ischemic damage, their activity within the brain is an indicator of neuronal integrity [22]. Previous studies in humans and animals have shown that irreversibly damaged cortical tissue can be detected by decreased binding of carbon-11 (^11^C) labeled FMZ [21,22]. FFMZ is a fluorinated analogue of flumazenil with similar pharmacokinetics. FFMZ can be labelled with fluorine-18 (^18^F), which is advantageous because of the longer half-life [23]. Previous studies have shown that PET imaging with ^18^F-FFMZ can be used to map the activity of GABA-A receptors in the human brain in the same way as ^11^C-FMZ [24].

^18^F-FFMZ-PET imaging was performed on the GE Healthcare Discovery VCT PET/CT scanner in the 3D acquisition mode. 370MBq of the tracer was injected 25min before the start of the PET imaging (160min after first ET-1 injection). A CT scan was acquired for attenuation correction. For the PET imaging, 5 frames of 300s duration each (25min total time) were acquired on 47 3.3mm thick slices. The 5 frames were averaged together at all slice locations.

### Data Analysis

**Fig 1:**
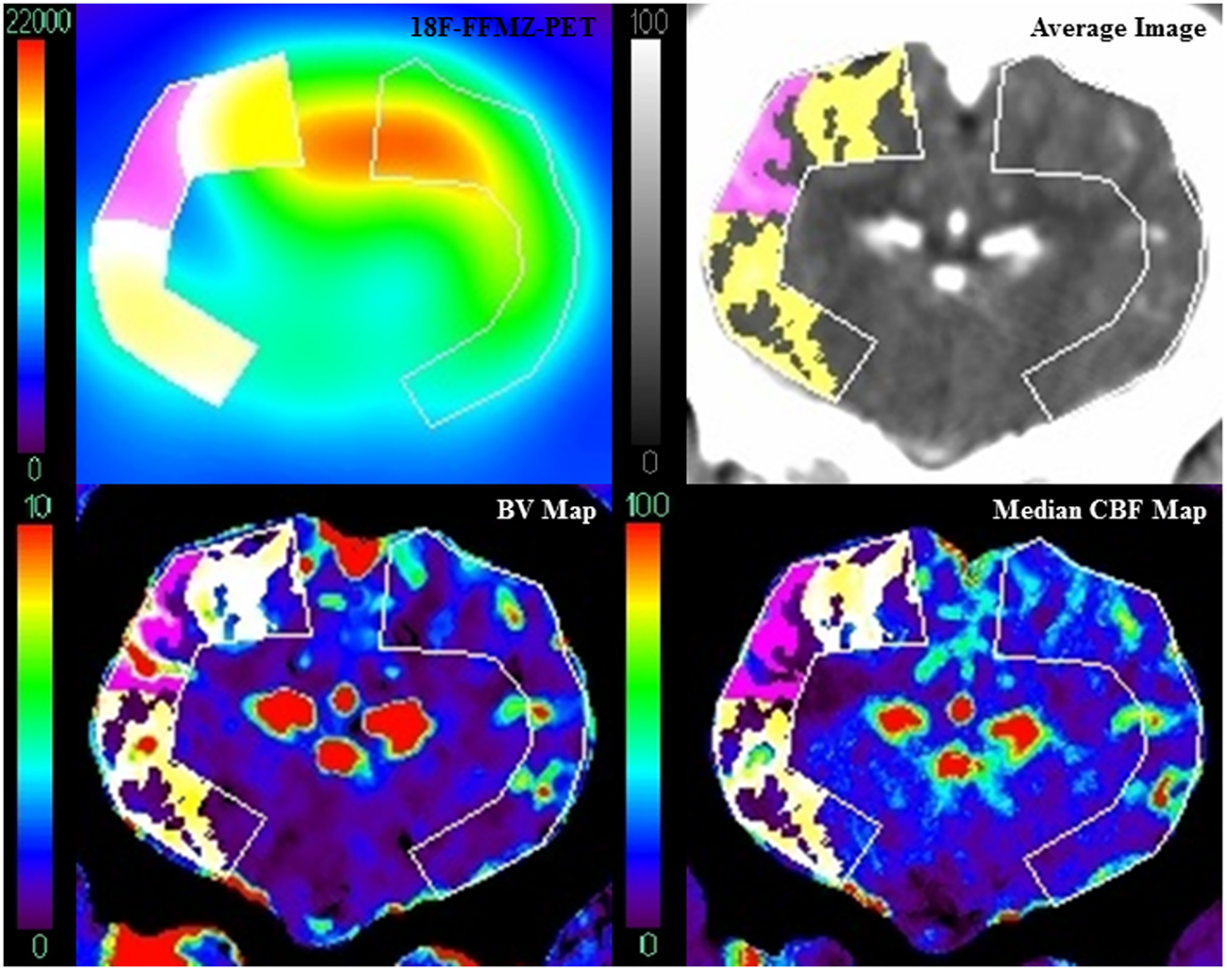
Image Analysis Method. Infarct (pink) was identified on a PET image (top left) acquired 160-185min after ET-1 injection as pixels in the affected side ROI with signal below the infarction threshold derived from the contralateral ROI. Pixels with signal above this threshold were classified as non-infarct (yellow). The average image (top right) was used to segment out white matter. Blood vessels were identified on the BV map (bottom left) using a threshold derived from the affected side ROI (see text). Grey matter, vessel-less infarct and non-infarct ROIs were then superimposed onto the median CBF map (bottom right).

For consistency, all CTP functional maps were calculated by one author using delay-insensitive deconvolution software (CT Perfusion 5 GE Healthcare, Waukesha, WI) as described previously [25]. For each pig, CBF maps from each CTP imaging time point were co-registered, and the median value of each pixel was found using Matlab, to generate a median CBF map. The median CBF map was then co-registered with the PET images, average images (perfusion-weighted maps) from the baseline CTP study, and blood volume (BV) maps at 10min after the first ET-1 injection. All image registration was manually performed with rigid 3D registration in Analyze 11 (Mayo Clinic, Biomedical Engineering). The average image was used to draw regions of interest (ROIs) covering the cortex on the affected and contralateral sides on all slices containing a defect in ^18^F-FFMZ uptake. These ROIs were then superimposed onto all other co-registered images. Infarct pixels were identified on PET images as having signal less than the average minus 2 standard deviations from the contralateral ROI. Since GABA-A receptors are located primarily in the grey matter [26], the average image was used to segment out white matter by removing any pixels where the CT number was less than 40 HU. To avoid the influence of large blood vessels on parenchymal perfusion, the BV map was used to exclude blood vessel pixels if they had a BV greater than the average plus 2 standard deviations from the affected side ROI. Additionally, pixels with median CBF greater than 100 mL·min^-1^·100g^-1^ were also considered to be blood vessels and excluded from analysis. The remaining infarct and non-infarct grey matter ROIs were then superimposed onto the median CBF map and pixel values were imported into an in-house Matlab program for logistic regression and ROC analysis (Fig 1). This process was repeated for each animal that had a defect in ^18^F-FFMZ uptake. The CBF values that corresponded to the optimal operating point of the ROC curve [27] for each animal were averaged together to determine the CBF threshold for infarction after 3hrs of ischemia.

## Results

6 out of 11 animals had irreversible tissue damage (i.e. uptake defect upon ^18^F-FFMZ imaging) and were included in the analysis. After removing vessel pixels, the volumes of grey matter infarct determined by the PET threshold for these animals were 2.96, 0.74, 2.30, 0.96, 0.97, and 0.80mL, giving an average grey matter infarct volume of 1.46 ± 0.38mL. 3 of the 6 animals that developed infarction required a second ET-1 injection to maintain depressed CBF in the ischemic territory.

**Fig 2:**
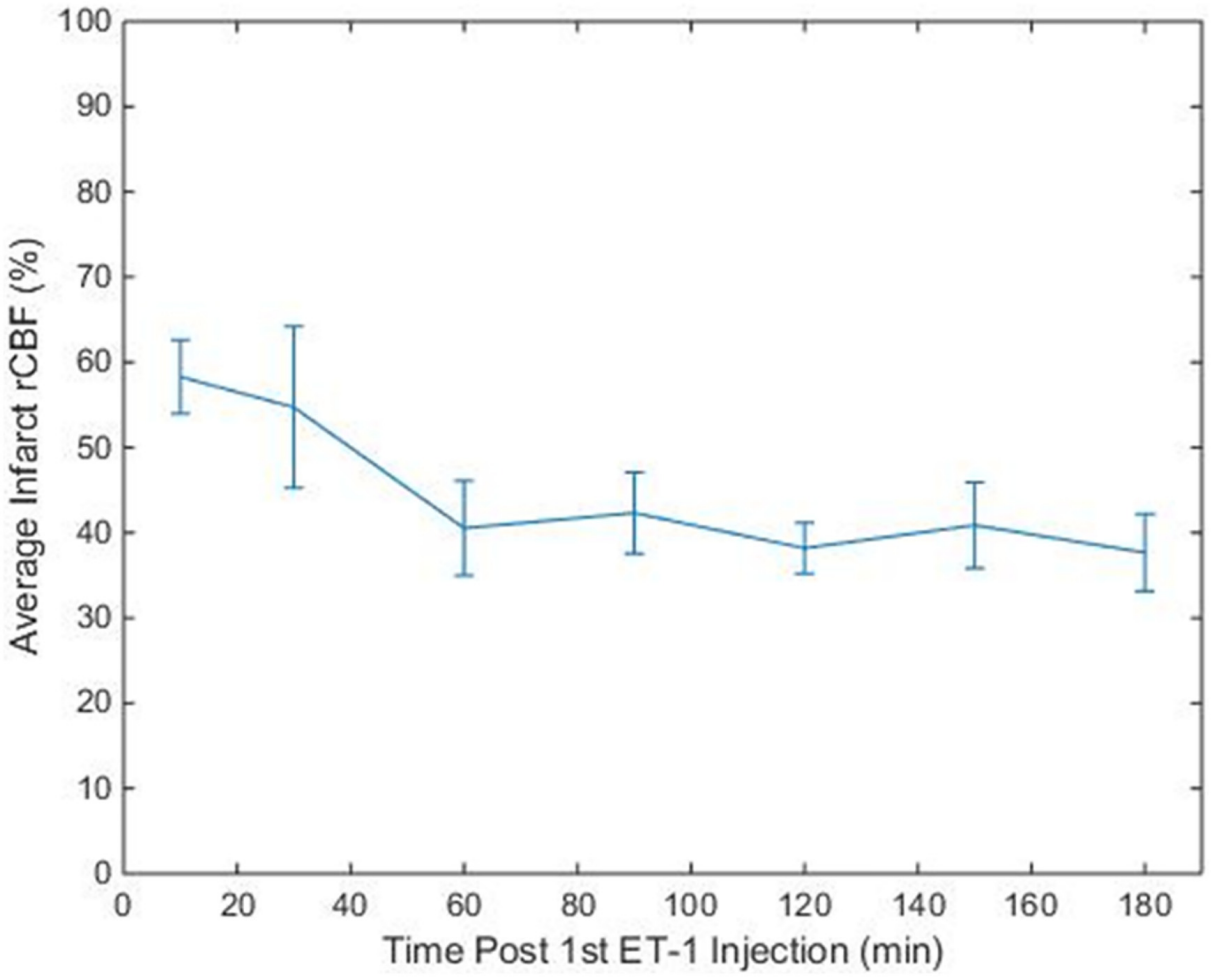
Average Relative CBF of Infarct ROIs. Average rCBF value from the infarct regions of the 6 animals at each time point. Error bars indicate standard error.

The average relative CBF (rCBF normalized to contralateral grey matter) in the grey matter infarct region was calculated at each CTP imaging time point, for each animal. The average relative CBF value in the infarct regions over all animals and CTP imaging time points was 42 ± 16%. Fig 2 shows the average rCBF in the infarct region over time. On average, 60min after the 1^st^ ET-1 injection rCBF dropped to ~40% and remained there for the duration of the experiment.

**Fig 3:**
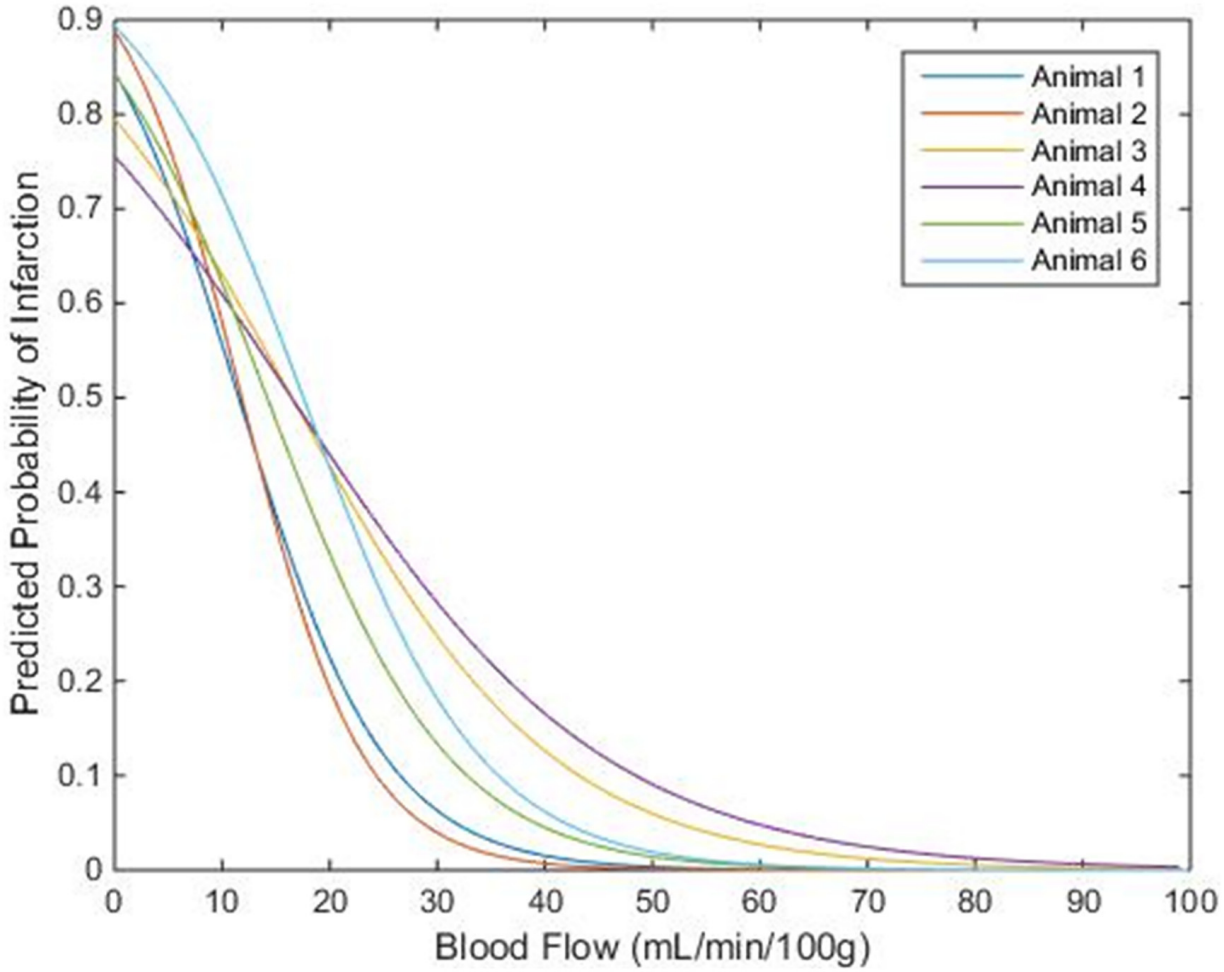
Predicted Probability of Infarction vs. CBF. Predicted probability of infarction from logistic regression plotted against CBF for each animal. The average of the CBF values that corresponded to a 75% predicted probability of infarction was approximately 4.5 ± 2.6 mL·min^-1^·100g^-1^.

The infarct CBF histograms for each animal were normalized by scaling the number of infarct pixels in each bin up by the ratio of the total penumbra/oligemia pixels to the total infarct pixels (this scaling operation equalized the number of pixels in the infarct and penumbra/oligemia histograms). This normalization did not affect the ROC analysis because the relative frequencies of the CBF values were not changed, but it was necessary to prevent bias in the logistic regression caused by having a much greater number of pixels in the penumbra/oligemia group than in the infarct group. The CBF histograms for each animal can be found in the S1 Fig. Matlab was used to perform a binary logistic regression on the normalized histogram data for each animal. The probability of infarction predicted by logistic regression is plotted against CBF for each animal in Fig 3. The average of the 6 CBF values that corresponded to a 75% predicted probability of infarction in each animal was 4.5 ± 2.6 mL·min^-1^·100g^-1^.

**Fig 4:**
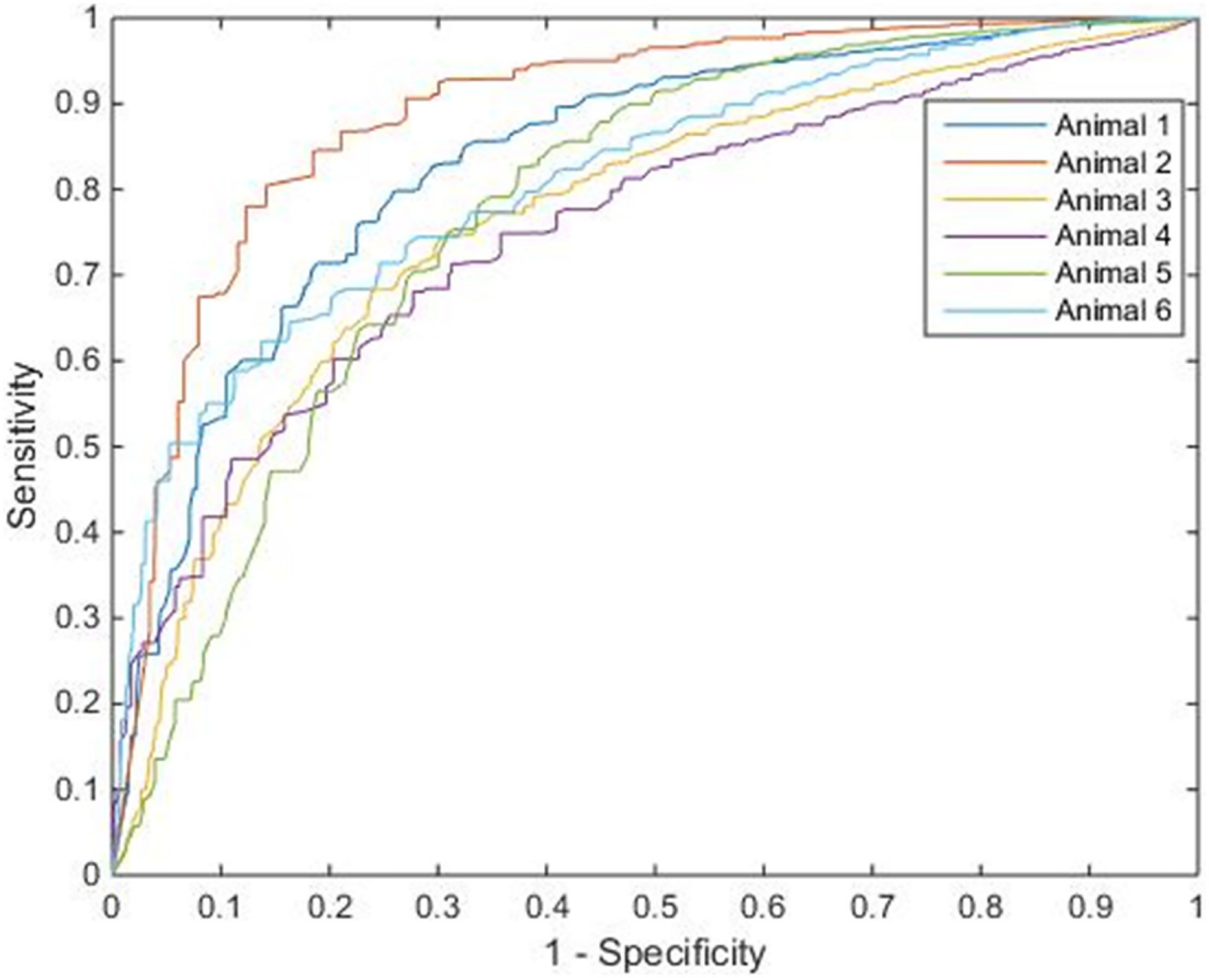
ROC Curve with Optimal Operating Point. ROC curves plotted for each of the 6 animals. Table 1 lists the CBF threshold derived from the optimal operating point of the ROC curve for each animal, and the corresponding sensitivity, specificity, accuracy, and AUC.

Fig 4 shows the ROC curve for each animal. The average of the CBF values corresponding to the optimal operating points of the ROC curves [27] was 12.6 ± 2.8 mL·min^-1^·100g^-1^. The sensitivity, specificity, and accuracy for infarct detection corresponding to the threshold in each animal, and the area under curve (AUC) can be found in Table 1.

**Table 1:** ROC Parameters for Each Animal.

Animal	Threshold (mL·min^-1^·100g^-1^)	Sensitivity	Specificity	Accuracy	Area Under Curve
1	10.1	0.80	0.74	0.77	0.8333
2	8.9	0.80	0.86	0.83	0.8908
3	15.0	0.74	0.70	0.72	0.7650
4	13.2	0.68	0.72	0.70	0.7528
5	12.1	0.75	0.69	0.72	0.7750
6	16.2	0.71	0.75	0.73	0.8110

The relevant parameters from the ROC analysis for each animal. The CBF threshold for infarction was the CBF value that corresponded to the optimal operating point of the ROC curve.

## Discussion

This study used a porcine model of ET-1 induced focal cerebral ischemia to determine a CTP-derived CBF threshold for infarction as determined by ^18^F-FFMZ-PET imaging after 3 hours of ischemia. A threshold of 12.6 mL·min^-1^·100g^-1^ was determined using ROC analysis. Previous animal studies have shown that the threshold for infarction is time dependent, dropping from 17–24 mL·min^-1^·100g^-1^ for permanent occlusion to 12 mL·min^-1^·100g^-1^ for occlusion lasting only 3 hours [20,28].

CTP-based time dependent thresholds for infarct core could be important for identifying late presenting or wake-up ischemic stroke patients that may still benefit from therapy [1]. Furthermore, time-dependent thresholds for infarction could be used to predict infarct growth in the time between admission imaging and reperfusion. This knowledge would be useful when deciding whether it is worthwhile to transfer a patient from a regional hospital to an IAT capable tertiary care hospital.

Prior perfusion threshold derivation studies could be affected by methodological problems. Many studies measure CBF during the acute phase of stroke, but do not determine the tissue outcome until several days or even weeks later [3–5] introducing the uncertainty of infarct expansion in the interim. Furthermore, some studies use sub-optimal imaging data sets where the degree of reperfusion is not known [4]. With the experimental procedure used in this study CBF measurements and tissue outcome can be determined contemporaneously, eliminating the error caused by infarct expansion. This model also allows consistent monitoring of CBF using CTP, which gives information about the extent of reperfusion in the ischemic tissue.

The experimental model used in this study has several advantages over other animal models. The model used by Jones et al involved surgery to expose the MCA so it could be occluded using a ligature [28]. This is an example of a broader category of models which induce ischemia by occluding the MCA, generally using either an intra-arterial catheter [29] or by exposing the MCA and applying a clip or ligature [28]. Implementing these models of acute ischemia in pigs can be problematic for several reasons; the rete mirabelle makes it impossible to use an intra-arterial catheter [14], and using a clip or ligature requires very invasive surgical procedures such as an osteotomy on the orbital rim [15] or removal of an eye [16]. The ET-1 insult used in this study circumvents these difficulties since the only surgeries required are an incision on the scalp and a small burr hole in the skull (~2mm diameter). The less traumatic approach increases the ability to maintain the animal at its basal physiological state throughout the experiment. This is supported by the fact that in the Jones study 13 of the 33 monkeys had to be excluded from the data analysis because of subarachnoid hemorrhage, problems with the ligature or other technical issues [28] whereas in this study none of the 11 animals experienced these problems.

In animals 1, 2, and 6 the first dose, administered at the start of the experiment, was able to maintain rCBF in the final infarct region below 50% for most of the experiment. However, in animals 3, 4, and 5 the effect of the first dose was transient and a second dose was required. The second dose was given 90min after the first dose in animal 4 and 30min after the first dose in animal 5, the average rCBF for the remainder of the experiments dropped to ~37% and ~32% respectively in the final infarct regions. In animal 3, the second dose was given 90min into the experiment, causing a transient decrease in rCBF to 43% before it rose above 50% again 150min into the experiment. In this animal the second dose may have been given too late to counteract the reactive hyperemia associated with reperfusion [30,31]. There was some variation in response to ET-1 injections, but variation in CBF reduction is to be expected in animal models of stroke. In the study by Jones et al where cerebral ischemia was initiated in monkeys by ligating the MCA, the average CBF after ligation in the insular cortex was 17 ± 16 mL·min^-1^·100g^-1^ [28], in this study the average CBF across all animals and time points after the first ET-1 injection was 15 ± 5 mL·min^-1^·100g^-1^. On average rCBF in the final infarct tissue dropped to 56% for the first 30min of the experiment, from the 1hr time point until the end of the experiment rCBF was maintained at ~40%. Although the cerebral ischemia caused by ET-1 injections does not always replicate clinical cases of stroke, the model is still suitable for this study, where the objective is to cause a reduction in CBF leading to infarction, then determine a CBF threshold for distinguishing salvaged tissue from tissue which progressed to infarction.

Reperfusion and subsequent reactive hyperemia of the ischemic tissue after the first ET-1 injection is problematic in this model for several reasons. It can stop ischemic tissue from progressing to infarction, in these experiments 5 of 11 animals did not develop irreversible tissue damage, and the main reason for this was premature reperfusion of the ischemic tissue. When reperfusion occurs midway through the experiment it can be difficult to accurately define the ischemia duration. Lastly, some tissue progresses to infarction despite having a relatively high CBF at many time points during the experiment, either due to a weak response to the first injection or premature reperfusion and associated reactive hyperemia after the first injection wears off. This can result in high median CBF in the infarct regions leading to overestimation of the infarction threshold. For example, the average rCBF in the final infarct region for animal 6 was below 30% between the 30min and 120min time points and above 40% in the final 3 time points. It is possible that infarction was due to CBF dropping to an average value of 9.4 mL·min^-1^·100g^-1^ for 30-90min in the middle of the experiment, and the higher CBF at the other time points resulted in an overestimated threshold of 16.2 mL·min^-1^·100g^-1^ being derived. Using the median CBF value rather than the average CBF lessens the effect that reperfusion has on the derivation of a threshold.

In our experiments, reperfusion was partially mitigated by using semi-continuous CBF monitoring with CTP at 30min intervals to identify when reperfusion had started, so that another dose of ET-1 could be given. At each CTP imaging time point the CBF maps were calculated on a work station in the CT scanner suite within ~5-8min of completing the scanning, to monitor for reperfusion from the wearing off of the ET-1 effects. This method effectively limited reperfusion of the ischemic tissue, as the average rCBF in the final infarct region was below 50% for the duration of the experiment in 5 of 6 animals, and the average infarct rCBF across all animals and time points was 42 ± 16%.

## Conclusions

The objective of this study was to determine a CBF threshold for infarction after 3 hours of ischemia. ROC analysis was used to find a threshold of 12.6 mL·min^-1^·100g^-1^, which agrees well with the value of 12 mL·min^-1^·100g^-1^ determined by Jones et al in 1981 [28]. The ET-1 model of acute stroke used in this study is easier to implement then other large animal stroke models. Despite some variation in response to ET-1 injections and instances of premature reperfusion, the model is comparable to other animal stroke models for the study objective.

## Supporting Information

S1 Fig Normalized frequency distributions for animals 1–6. (TIF)

S2 Fig Animal 1: PET image and CBF maps in slice with largest extent of infarct. (TIF)

S3 Fig Animal 2: PET image and CBF maps in slice with largest extent of infarct. (TIF)

S4 Fig Animal 3: PET image and CBF maps in slice with largest extent of infarct. (TIF)

S5 Fig Animal 4: PET image and CBF maps in slice with largest extent of infarct. (TIF)

S6 Fig Animal 5: PET image and CBF maps in slice with largest extent of infarct. (TIF)

S7 Fig Animal 6: PET image and CBF maps in slice with largest extent of infarct. (TIF)
