# Supplementary figures and images for: Absolute Cerebral Blood Flow Infarction Threshold for 3-Hour Ischemia Time Determined with CT Perfusion and 18F-FFMZ-PET Imaging in a Porcine Model of Cerebral Ischemia
Source: PLoS One. 2016 Jun 27;11(6):e0158157. doi: 10.1371/journal.pone.0158157 (PMC4922566; doi:10.1371/journal.pone.0158157)

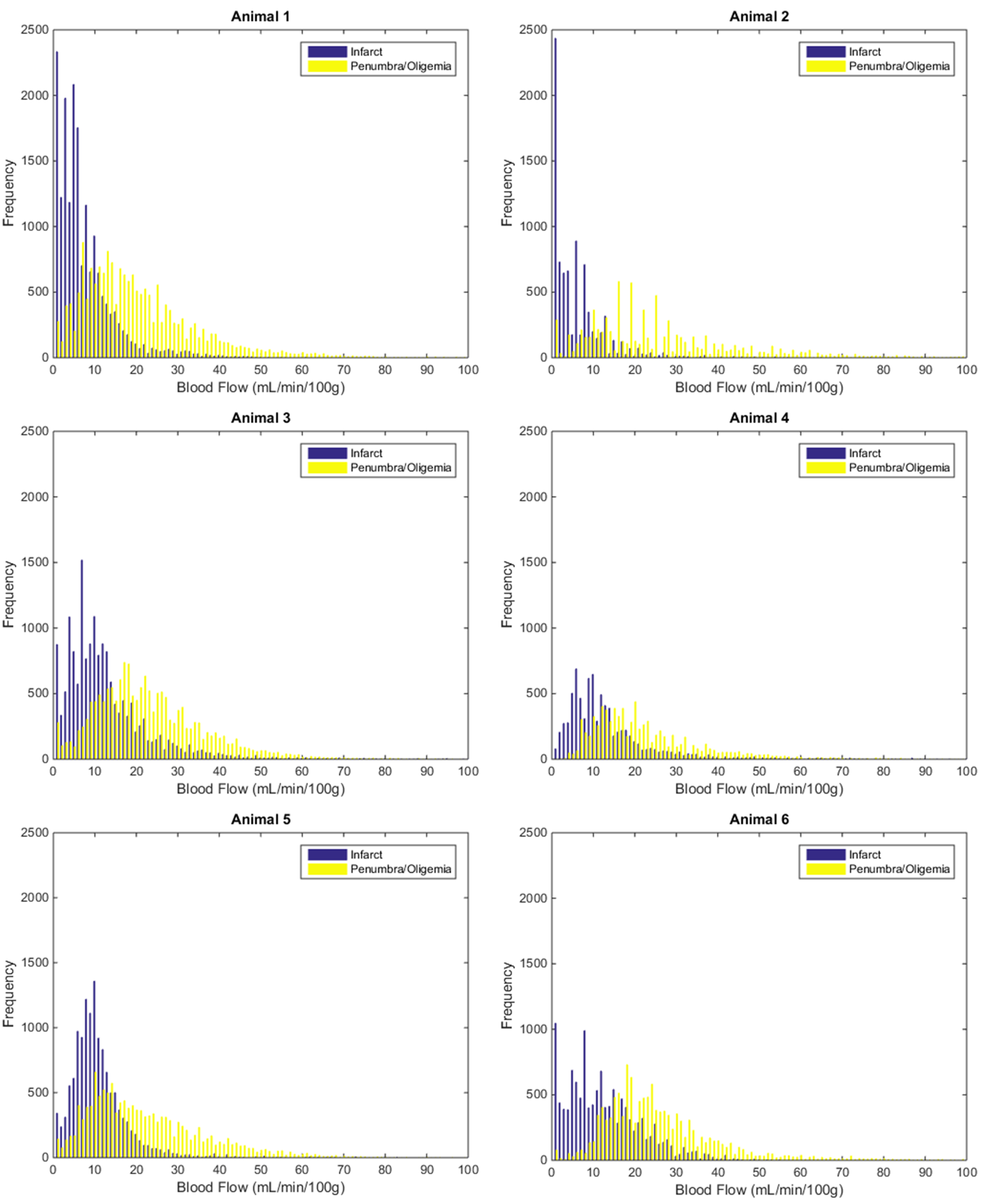

Supplement: S1 Fig — (TIF) [file pone.0158157.s001.tif]

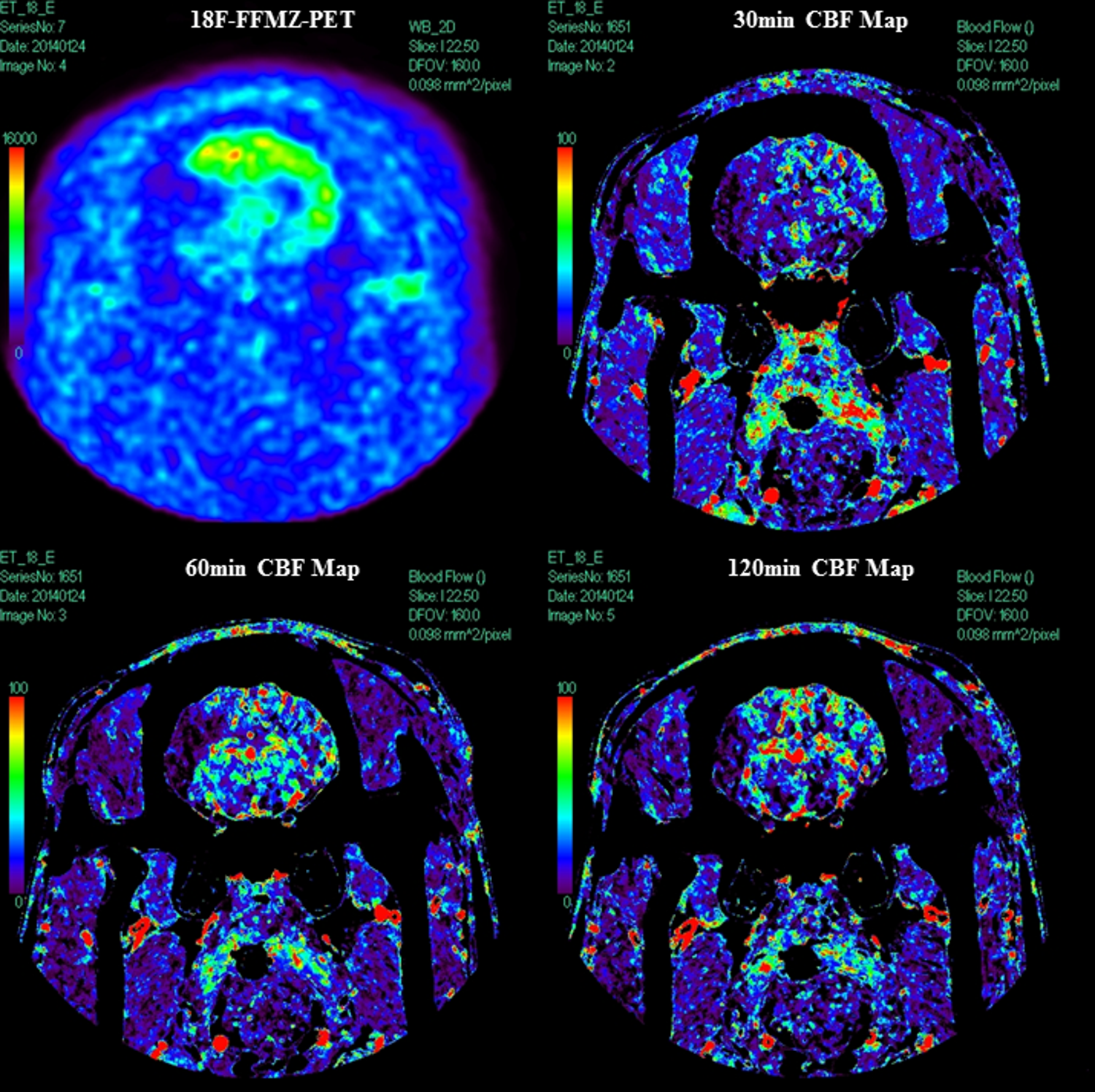

Supplement: S2 Fig — (TIF) [file pone.0158157.s002.TIF]

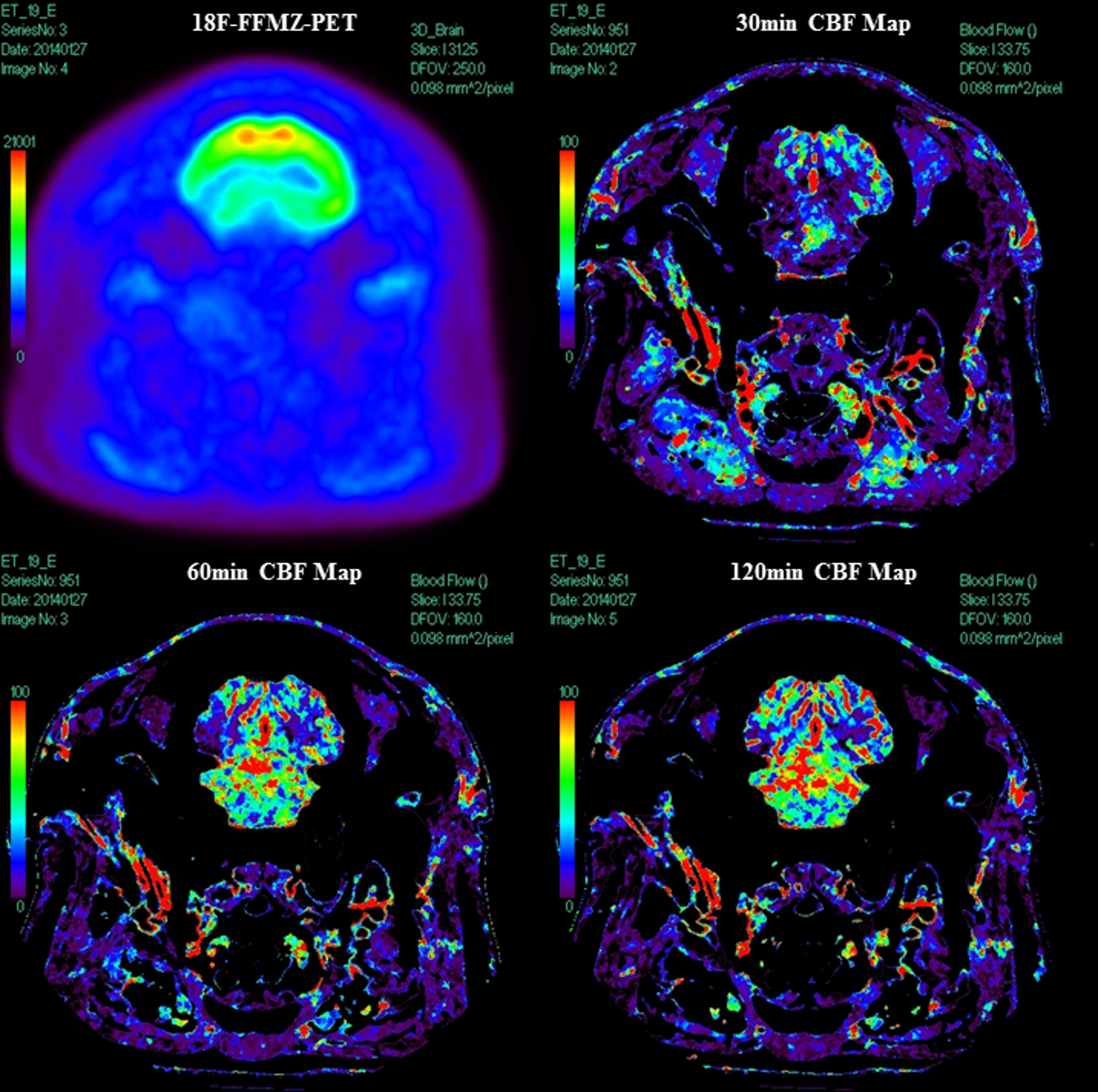

Supplement: S3 Fig — (TIF) [file pone.0158157.s003.TIF]

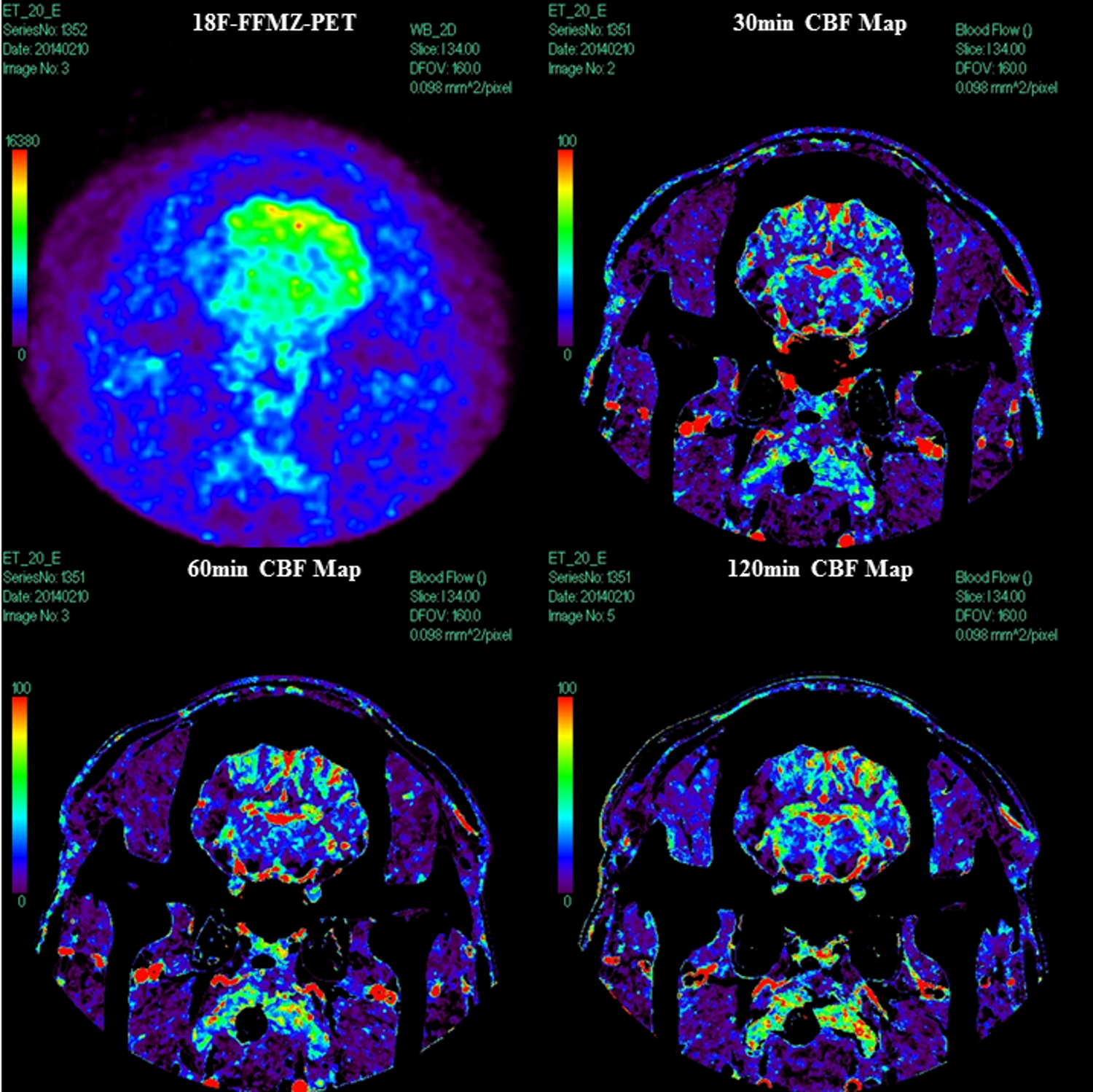

Supplement: S4 Fig — (TIF) [file pone.0158157.s004.TIF]

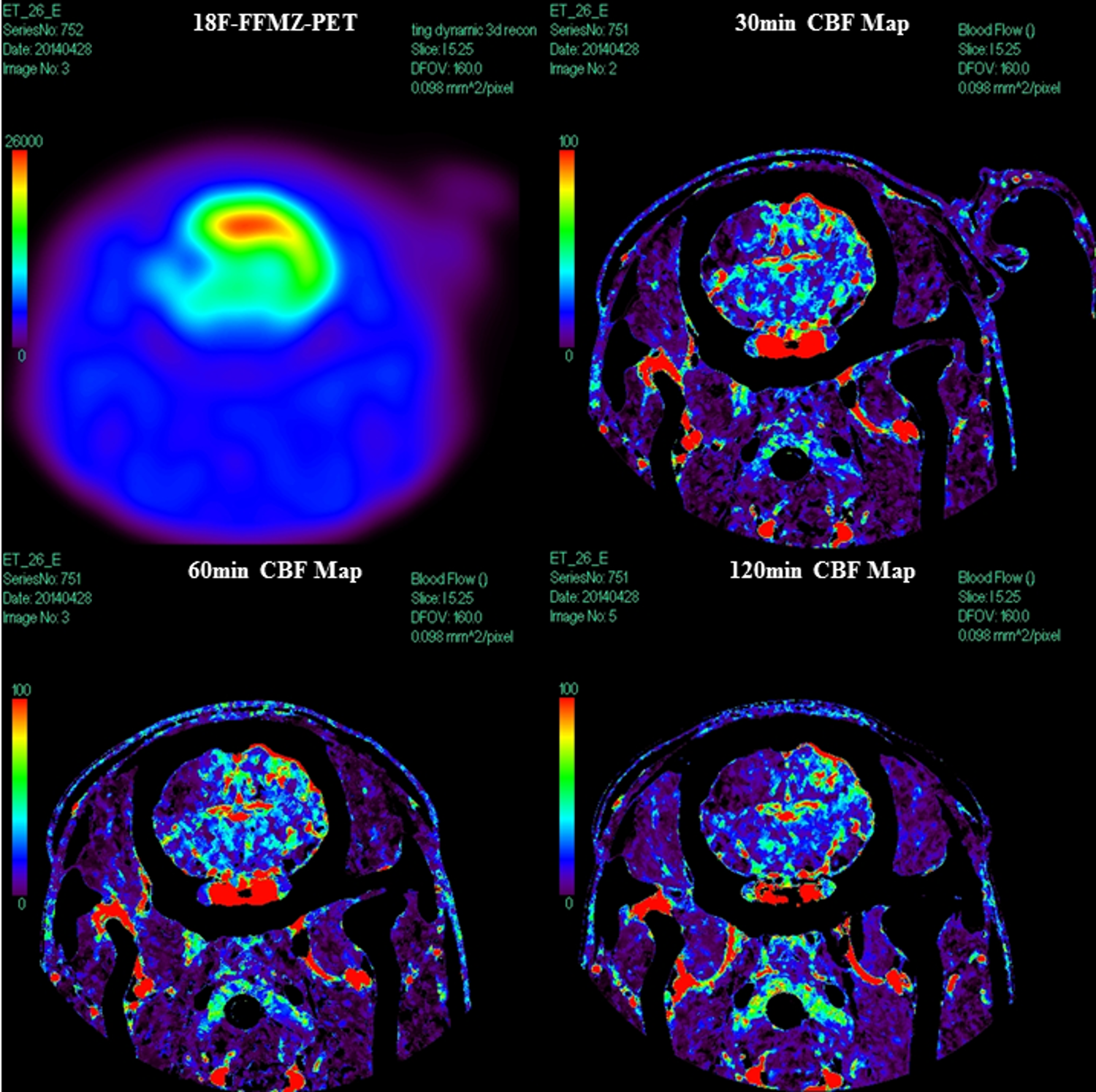

Supplement: S5 Fig — (TIF) [file pone.0158157.s005.TIF]

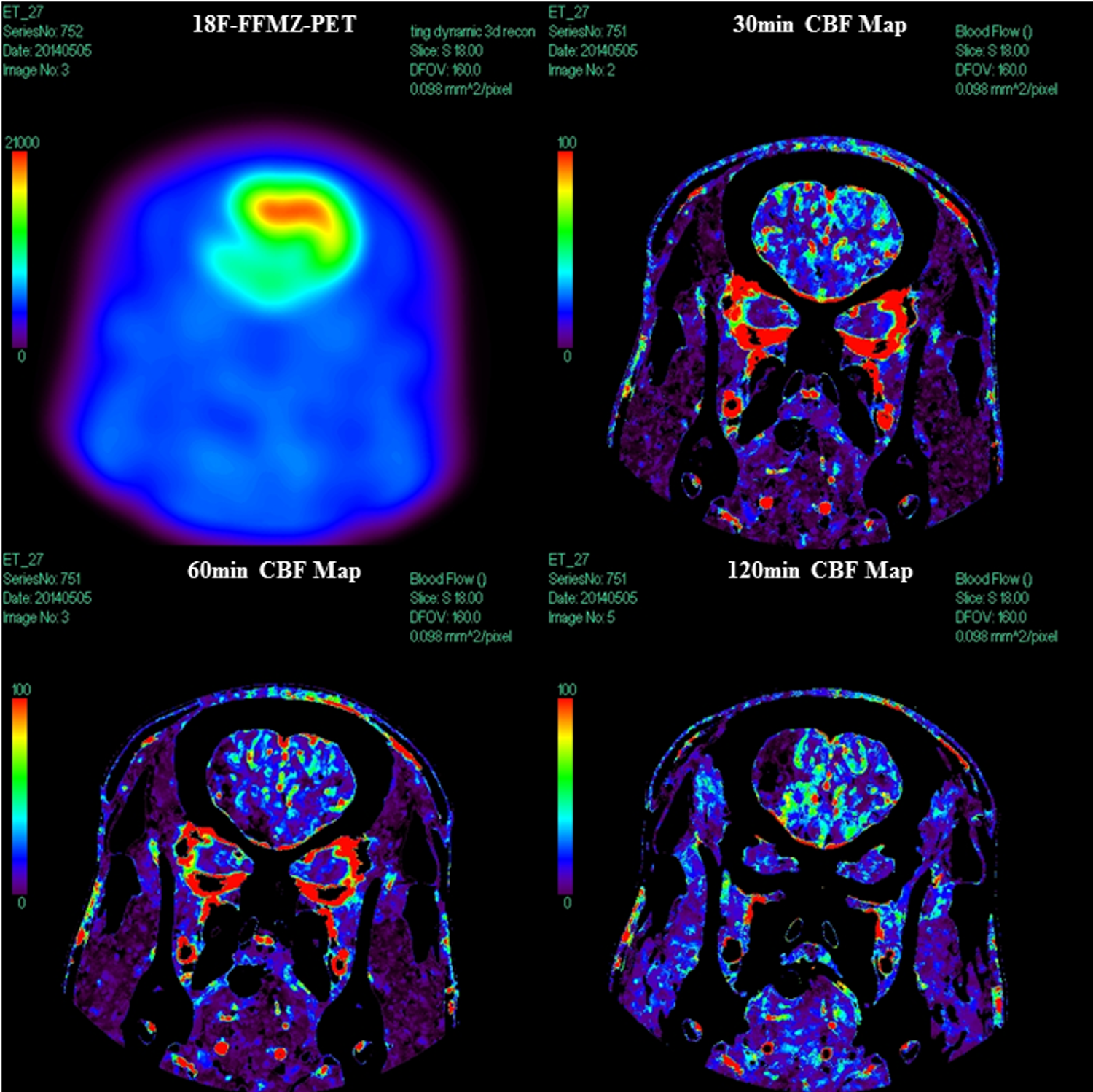

Supplement: S6 Fig — (TIF) [file pone.0158157.s006.TIF]

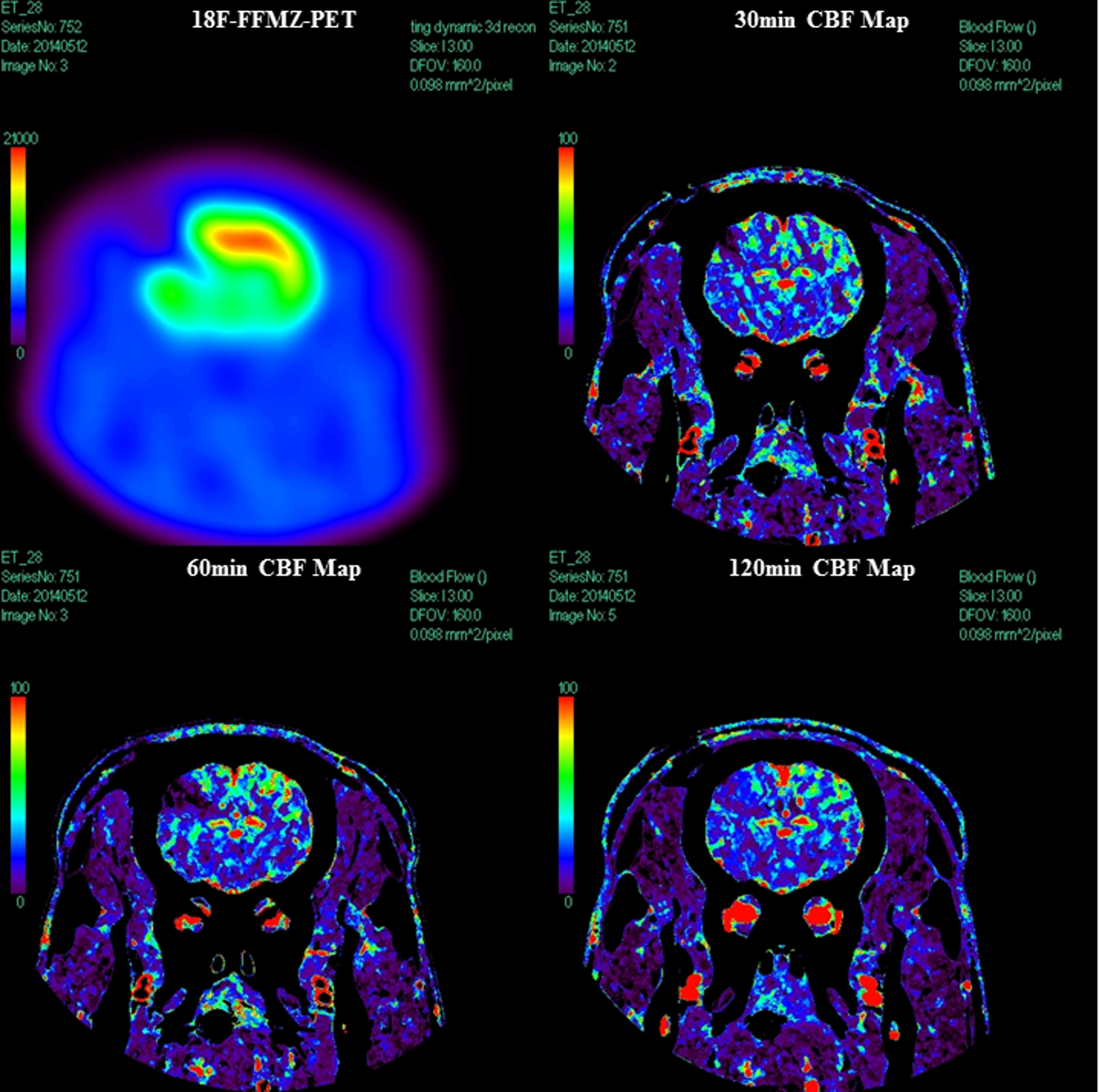

Supplement: S7 Fig — (TIF) [file pone.0158157.s007.TIF]
